# Risk and Prognostic Factors for Different Organ Metastasis in Primary Osteosarcoma: A Large Population‐Based Analysis

**DOI:** 10.1111/os.13243

**Published:** 2022-03-16

**Authors:** Guijun Xu, Haixiao Wu, Yanting Zhang, Yao Xu, Xu Guo, Vladimir P. Baklaushev, Vladimir P. Chekhonin, Karl Peltzer, Jun Wang, Feng Lu, Guowen Wang, Xin Wang, Wenjuan Ma, Chao Zhang

**Affiliations:** ^1^ Department of orthopaedics Tianjin Hospital Tianjin China; ^2^ Department of Bone and Soft Tissue Tumors, Tianjin Medical University Cancer Institute and Hospital, National Clinical Research Center for Cancer, Key Laboratory of Cancer Prevention and Therapy Tianjin's Clinical Research Center for Cancer Tianjin China; ^3^ Department of Orthopedics Cangzhou Central Hospital Cangzhou China; ^4^ Federal Research and Clinical Center of Specialized Medical Care and Medical Technologies Federal Biomedical Agency of the Russian Federation Moscow Russian Federation; ^5^ Department of Basic and Applied Neurobiology Federal Medical Research Center for Psychiatry and Narcology Moscow Russian Federation; ^6^ Department of Research and Innovation University of Limpopo Turfloop South Africa; ^7^ Department of Oncology, Radiology and Nuclear Medicine Medical Institute of Peoples' Friendship University of Russia Moscow Russian Federation; ^8^ Department of Epidemiology and Biostatistics, West China School of Public Health Sichuan University Chengdu China; ^9^ Department of Breast Imaging, Tianjin Medical University Cancer Institute and Hospital, National Clinical Research Center for Cancer, Key Laboratory of Cancer Prevention and Therapy Tianjin's Clinical Research Center for Cancer Tianjin China

**Keywords:** Metastases, Mortality, Osteosarcoma, Prognosis

## Abstract

**Objective:**

Based on a large public cohort, we aimed to investigate the prevalence of distant metastases in patients with osteosarcoma, to evaluate the survival of patients with different metastases and to reveal the related risk and prognostic factors for distant metastases.

**Methods:**

The information of osteosarcoma patients with or without distant metastases was retrospectively extracted from the Surveillance, Epidemiology, and End Result database from January 2010 to December 2015. Patients were excluded if they were diagnosed at autopsy or *via* death certification. The Kaplan–Meier method was used to calculate the overall survival in the entire cohort and across patients with metastases to different organs. The related prognostic factors were investigated by univariate and multivariate Cox proportional hazard regression analysis. The logistic regression method was used to reveal the risk factors for the development of different metastases. The effects of different variables on the survival and prevalence of distant metastases were compared using subgroup analysis. Variables with *P* < 0.05 in the univariate regression analysis were further examined using multivariate regression analysis.

**Results:**

In total, 1470 osteosarcoma patients (mean age 30 ± 22 years) were included, among which 278 patients (18.9%) were initially diagnosed with distant metastasis. The median follow‐up duration was 33.0 (30.2–35.8) months. The lung was the most common metastatic site (83.8%), followed by the bone (21.9%), liver (2.9%), and brain (2.2%). A total of 232 patients (83.5%) presented only one distant metastatic site, while the other 46 patients showed two or more metastatic sites. A lower proportion of metastasis was observed in patients aged from 25 to 59 years [odds ratio (OR) = 0.59; 95% confidence interval (CI): 0.37–0.95]. More metastases were noted in patients with T2/T1 (OR = 1.91; 95% CI: 1.28–2.84), T3/T1 (OR = 4.48; 95% CI: 1.78–11.30) and N1/N0 stages (OR = 6.66; 95% CI: 2.68–16.56). The 1‐, 3‐, and 5‐year overall survival rates for metastatic patients were 57.3% (95% CI: 50.8%–63.8%), 25.3% (95% CI: 18.8%–31.9%), and 18.1% (95% CI: 10.2%–26.0%), respectively. Metastatic patients older than 25 years were prone to have poor survival and a relatively better prognosis (hazard ratio = 0.41; 95% CI: 0.25–0.69) was noticed among those who underwent surgery on the primary site. Different metastatic organs have homogeneous and heterogeneous risk and prognostic factors.

**Conclusion:**

The high incidence of initial distant metastasis in osteosarcoma and the inconsistent predictive factors should be given more attention in the clinical management of patients with osteosarcoma.

## Graphical abstract

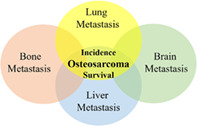

Homogenous and heterogenous risk and prognostic factors were noticed for osteosarcoma patients with distant metastasis to different organs, which can potentially guide organ‐specific distant metastasis screening and management.

## Introduction

Osteosarcoma is the most common type of bone cancer in children and young adults, with a reported annual incidence of 3–4 per million.^1^ Since the implementation of multiagent chemotherapy in the 1980s, the survival of patients with nonmetastatic osteosarcoma has significantly improved. According to the findings of 2260 patients from April 2005 to June 2011 in the European and American Osteosarcoma Studies, the 5‐year overall survival (OS) and event‐free survival from biopsy were 71% (68%–73%) and 54% (52%–56%), respectively.^1^ However, the prognosis of patients with distant metastasis remains poor.^2^ Compared with patients with localized osteosarcoma, the 5‐year OS of patients with metastasis was less than 30%.^3^ Distant metastasis has been recognized as a major issue in osteosarcoma management.

Because no significant symptoms in organs with metastasis can be observed at early stages, distant metastasis is difficult to be identified in a timely manner. Therefore, many cancer patients suffer distant metastases at the initial diagnosis.^4^, ^5^ The incidence of initial distant metastasis in osteosarcoma patients is approximately 15%,^6^ and the accurate prevalence of metastasis is thought to be underestimated. Cancer may be the most serious medical problem worldwide, and huge medical and monetary resources have been spent in the management of cancer prevention and treatment. Therefore, more attention should be given to patients with a high risk of metastasis to detect possible metastasis in a timely manner and to offer appropriate treatment.

As reported, more than 70% of metastatic sites involve the lung,^7^ and pulmonary metastasis significantly reduces patient survival. The 5‐year OS and disease‐free survival after pulmonary metastasis were 30% and 21%, respectively.^8^ The survival of patients with osteosarcoma post‐pulmonary metastasis is 16.0 months.^9^ An increasing number of studies have been conducted on pulmonary metastasis in osteosarcoma, including diagnosis,^10^ surgery,^8^ irradiation^11^ and prognosis.^12^ Improved survival in patients with pulmonary metastasis can be achieved by metastasectomy^13^ in those after completion of chemotherapy^14^ and in patients with fewer lung lesions. Lung CT is regularly recommended in osteosarcoma, and the related risk factors are indicative of patients with potential pulmonary metastasis. Due to the negative influence of metastasis on survival, determining prognostic factors could help clinicians tailor targeted treatment and improve survival.

Metastasis to other organs has also been reported among patients with osteosarcoma. A previous study reported abdominal metastasis in 2/94 patients (2.1%) with osteosarcoma.^15^ Some cases have also been reported to involve hepatic,^16^ pancreatic^17^ and renal metastasis.^18^ Metastatic sites have also been found in the brain,^19^ bone^20^ and heart.^21^ These rare sites are mostly identified by accident. No similar study could be found in recent decades, and no regular screening was suggested in the latest National Comprehensive Cancer Network guideline for bone cancer. In addition, limited by small sample sizes, few studies have investigated the clinical features and prognostic factors among patients with other sites of distant metastasis. Limited risk factors have been reported in previous studies. Therefore, further identification of key factors associated with different metastases is needed for metastasis prediction and survival improvement.

Established in 1973, the Surveillance, Epidemiology, and End Result (SEER) database (https://seer.cancer.gov/data/) has been collecting data from 18 cancer registries and comprises approximately 30% of the total US population. A large sample size of osteosarcoma patients permitted the comprehensive study of distant metastases. In our previous studies based on the SEER database, osteosarcoma^22^ patients with initial pulmonary metastasis^9^ were examined. A series of significant parameters were found to be correlated with metastasis prediction and survival estimation. Axial location, tumor size >10 cm (odds ratio, OR = 3.195), higher N stage (OR = 4.84) and presence of bone metastases (OR = 8.73) or brain metastases (OR = 25.63) were significantly associated with lung metastases.^9^ In the present study, based on a large cohort of osteosarcoma patients from the SEER database, we aimed to investigate the latest prevalence and survival conditions of osteosarcoma patients with different metastases. We further (i) examined the patterns of distant metastasis in osteosarcoma and (ii) investigated the risk and prognostic factors of osteosarcoma patients with different distant metastases.

## Patients and Methods

### Inclusion and Exclusion Criteria

The inclusion criteria were as follows: (i) patients with osteosarcoma; (ii) clear information of the metastatic sites and surgery treatments could be extracted; (iii) vital status and survival time were recorded; and (iv) related variables were complete. Patients were excluded if they were diagnosed at autopsy or by death certification. Patients with incomplete records of distant metastasis were also excluded.

### Data Source and Cohort Selection

**Fig. 1:**
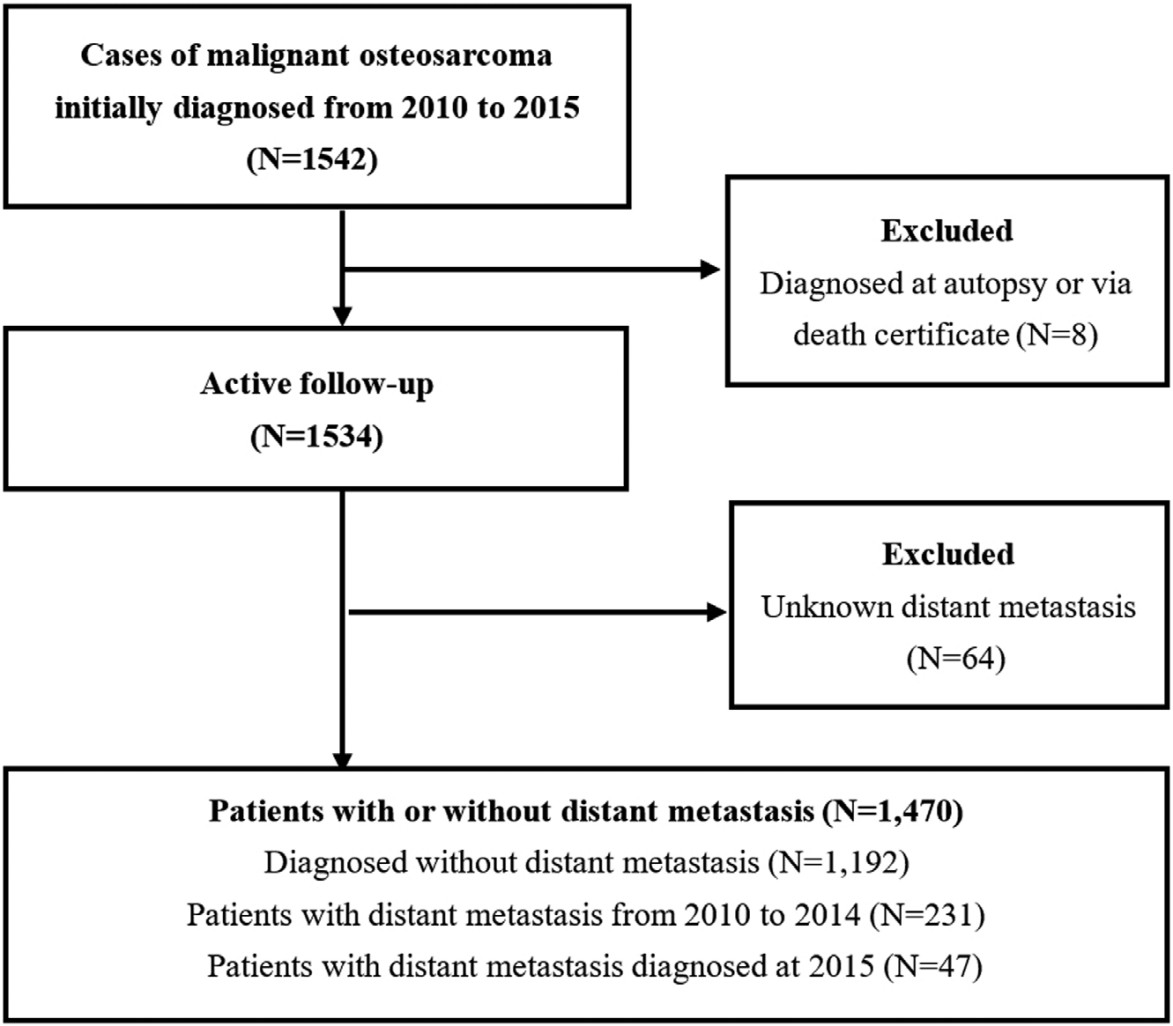
Flowchart of patient selection from the SEER database. Records from January 2010 to December 2014 were used for survival estimation and prognostic factor evaluation. Records from January 2010 to December 2015 were analyzed to identify risk factors for distant metastasis.

Based on the SEER database, patients who were diagnosed with osteosarcoma were included. Since the information of metastatic sites was not recorded before 2010, we limited osteosarcoma patients to those diagnosed between January 2010 and December 2015. Figure 1 illustrates the selection of the possible subjects. To analyze the prognostic factors of osteosarcoma patients with distant metastasis, patients diagnosed between January and December 2015 were excluded. Eventually, 231 patients diagnosed between January 2010 and December 2014 were included for further survival analysis.

### Outcome Measures

According to the status of distant metastasis, patients were subgrouped into patients without distant metastasis and patients with specific metastatic sites.

#### Prevalence and Risk Factors

The prevalence was the ratio of the occurrence of distant metastasis to the number of populations at risk for a time period. The risk factors were those significantly associated with the occurrence of distant metastasis as revealed by the univariate and multivariate logistic regression model.

#### Overall Survival and Prognostic Pactors

OS was used to indicate the time from diagnosis to death by all causes. For subjects who were lost to follow‐up prior to death, the last follow‐up time was used as the time of death.

The prognostic factor was defined as that related to the survival of patients and could be used for prognosis prediction. The factors were identified by Cox proportional hazard regression analysis.

### Statistical Analysis

Pearson's chi‐square test and rank‐sum test were used to determine the significant differences in demographic and clinicopathological variables in the total cohort and different metastatic groups. Univariate logistic regression analysis was initially performed to analyze the risk factors for distant metastasis development. Factors with *P* < 0.10 and other important factors based on doctors' experience were further analyzed by multivariate logistic regression. In the survival analysis, surgical treatment was further added. The primary outcome of the survival analysis was OS. Survival analysis was obtained using the Kaplan–Meier method; the differences between the survival curves were tested by the log‐rank test. The OS difference between the patients with or without distant metastasis was calculated by subgroup analysis. Univariate and multivariate Cox regression analyses were then performed to investigate independent prognostic factors.

SEER*Stat Software version 8.3.5 (https://seer.cancer.gov/seerstat/) was used to obtain all of the data. All statistical analyses were performed using SPSS 22.0 (IBM Corporation, Armonk, NY, USA), and all charts on survival were prepared by MedCalc 18.11.3 (MedCalc Software Ltd., Acacialaan, Ostend, Belgium). The statistical significance level was set as a two‐sided *P value* < 0.05.

## Results

### General Results

According to the predefined inclusion criteria, 1470 osteosarcoma patients were initially identified in a median follow‐up of 33.0 (30.2–35.8) months. Among them, 278 (18.9%) patients showed distant metastases. Males were predominantly affected, accounting for 54.89% of all patients in the cohort. The mean age was 30 ± 22 years, and 57.3% of patients were younger than 25 years. Regarding the tumor sites, the extremities occupied 72.3% of patients compared with others in the axial body. The main histological subtype was osteosarcoma NOS 977 (66.5%). Patients at grade IV accounted for 42.6% of all included patients, followed by grade III (357, 24.3%). In total, 83.6% of the osteosarcoma patients received surgical treatment. The patients' characteristics are summarized in Supplementary Table S1.

### Distant Metastatic Patterns and Risk Factors

Overall, 278 osteosarcoma patients were diagnosed with distant metastasis. The incidence of distant metastasis in osteosarcoma ranged from 17% in 2010 to 22% in 2012. The proportion of osteosarcoma NOS subtype was 75.2%. The lung was the most common metastatic site (233, 83.8%), followed by bone (61, 21.9%), liver (eight, 2.9%), and brain (six, 2.2%). A total of 232 patients (83.5%) presented only one distant metastatic site, while the other 46 patients showed two or more metastatic sites.

Univariate logistic regression identified the risk factors for the development of all distant metastases, including patients older than 60 years [OR = 1.44; 95% confidence interval (CI): 1.01–2.05; *P* = 0.043)], tumor grade III (OR = 16.22; 95% CI: 2.21–118.94; *P* = 0.006) and IV (OR = 14.04; 95% CI: 3.50–14.58; *P* = 0.009), stage T2 (OR = 2.33; 95% CI: 1.67–3.25; *P* < 0.001) and T3 (OR = 7.14; 95% CI: 3.50–14.58; *P* < 0.001), and N1 stage (OR = 6.78; 95% CI: 3.28–14.03; *P* < 0.001). Fewer distant metastases were diagnosed in female patients (OR = 0.70; 95% CI: 0.54–0.92; *P* = 0.01) and patients aged between 25 and 59 years (OR = 0.67; 95% CI: 0.49–0.93; *P* = 0.017). More information about the univariate logistic regression for different metastases is shown in Supplementary Table S2.

Multivariate analysis (Supplementary Table S3) revealed less metastasis in patients aged 25 to 59 years (*vs* age < 25 years) (OR = 0.59; 95% CI: 0.37–0.95; *P* = 0.028). More metastases were found in higher T grades T2 (OR = 1.91; 95% CI: 1.28–2.84; *P* = 0.001) and T3 (OR = 4.48; 95% CI: 1.78–11.30; *P* = 0.001) and N1 stage (OR = 6.66; 95% CI: 2.68–16.56; *P* < 0.001). Patients older than 60 years showed a trend similar to those younger than 25 years. A comparable influence was noticed in sex and different histological types. The risk factors were not consistent when stratified by different metastatic organs. Patients at the N1 stage showed a relatively high proportion of distant metastasis in the lung (OR = 5.81; 95% CI: 2.34–14.45; *P* < 0.001), liver (OR = 13.98; 95% CI: 1.44–135.98; *P* < 0.001) and bone (OR = 9.09; 95% CI: 3.32–24.90; *P* < 0.001). More lung and bone metastasis occurred for patients with higher T stage (T2, T3). Patients aged from 25 to 59 years were unlikely to develop lung metastasis, while patients with a primary site in the axial body were independently associated with bone metastasis. Patients with metastasis to more than one site were significantly associated with brain metastasis.

### Survival and Prognostic Factors of Osteosarcoma Patients with Distant Metastasis

**Fig. 2:**
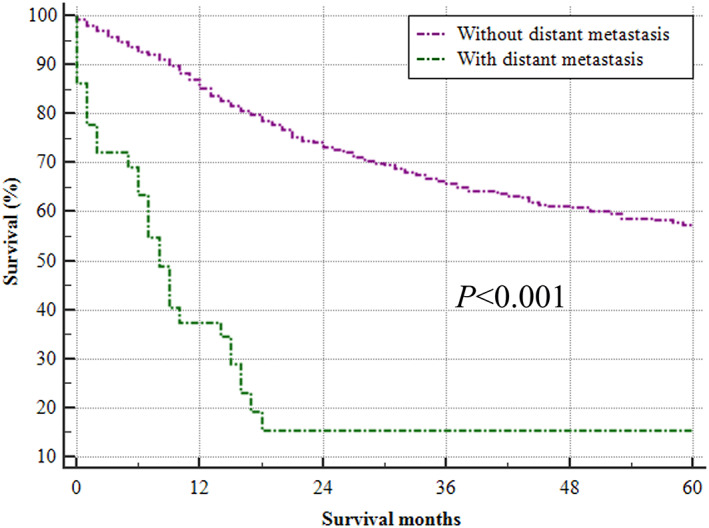
Kaplan–Meier analysis of overall survival for patients with distant metastasis (green line) or without distant metastasis (purple line) based on records from January 2010 to December 2014.

The survival of patients with or without distant metastasis is illustrated in Fig. 2. The 1‐, 3‐, and 5‐year OS rates for patients without metastasis were 85.1% (95% CI: 83.0%–87.1%), 65.7% (62.7%–68.7%), and 57.4% (53.6%–61.1%), respectively. In addition, the 1‐, 3‐, and 5‐year OS rates for patients with metastasis were 57.3% (50.8%–63.8%), 25.3% (18.8%–31.9%), and 18.1% (10.2%–26.0%), respectively. Detailed information on OS for each variable between patients with or without distant metastasis is summarized in Supplementary Table S4. Regarding different metastatic sites, the median OS for osteosarcoma patients with lung, bone, liver and brain metastasis was 17 (13–20), 10 (8–15), 1 (0–6), and 2 (1–9) months, respectively.

The results of univariate Cox regression analysis are shown in Supplementary Table S5. For the entire cohort, older age (≥25 years *vs* <25 years), marital status, primary site in the axial (*vs* extremity), and >1 metastatic site were negatively associated with survival. Both chondroblastic subtype (*vs* osteosarcoma NOS) and surgery at the primary site were correlated with improved OS. No significant factors were identified for patients with liver or brain metastasis.

Multivariate Cox regression analyses for all patients with metastasis, lung metastasis and bone metastasis were further performed, and the results are shown in Supplementary Table S6. Compared with patients younger than 24 years, older age [≥60 years, hazard ratio (HR) = 3.97, 95% CI: 1.99–7.91; *P* < 0.001), 24–59 years, HR = 1.96, 95% CI: 1.11–3.45, *P* = 0.020] was an independent prognostic factor leading to poor survival in patients with distant metastasis, while surgery on the primary site improved survival (HR = 0.41, 95% CI: 0.25–0.69, *P* = 0.001). When stratified by specific metastatic organs, female sex was an independent prognostic factor for bone metastasis (HR = 3.85, 95% CI: 1.02–14.51, *P* = 0.047), while age older than 60 years (compared with patients younger than 25 years) was an independent prognostic factor for lung metastasis (HR = 3.07, 95% CI: 1.37–6.88, *P* = 0.007). In addition, surgery on the primary site was a protective factor for patients with lung metastasis (HR = 0.30, 95% CI: 0.16–0.57, *P* < 0.001).

## Discussion

Osteosarcoma, representing over 50% of all bone tumors, was reported to have high metastatic potential. Approximately 80% of osteosarcoma patients eventually develop metastasis even after surgical treatment.^23^ Meanwhile, many osteosarcoma patients are initially diagnosed with metastasis. In the present study, 18.9% of all patients showed distant metastasis at the initial diagnosis. This prevalence of distant metastasis was in accordance with a previous systematic review, within which the prevalence was 15% and 20% in patients from regions with high and low human development index scores, respectively.^24^

### Patterns of Distant Metastasis in Osteosarcoma

Metastasis to the lung is the most common site in patients with osteosarcoma.^7^ Many studies have confirmed the negative influence of lung metastasis on the survival of osteosarcoma patients.^12^, ^25^ In our study, more cases with lung metastasis were found in patients with higher T stage (T2, T3) and N1 stage and those younger than 25 years or older than 60 years. Thus, more attention should be given to lung metastasis screening in osteosarcoma patients with the identified risk factors, and a higher frequency of lung CT scans should be scheduled for these patients. Bone was found to be the second most common metastatic site, occupying 21.9% of all metastatic sites. As reported, 14.3% of osteosarcoma patients were identified to have distant metastasis through whole‐body MRI.^26^ Metastases to the brain and liver in osteosarcoma have rarely been reported.^27^ The incidence of brain and liver metastasis in osteosarcoma was less than 3% in the present study. However, the survival of patients with brain or liver metastasis was significantly decreased compared with that of patients with lung or bone metastasis.

### Risk and Prognostic Factors for Distant Metastasis

Osteosarcoma patients with distant metastasis showed worse survival. In a previous study, the survival of patients with primary metastatic osteosarcoma was significantly correlated with age, primary tumor site, response to neoadjuvant chemotherapy, numbers and sites of metastasis, and surgical resection of the tumor sites.^28^ In the present study, based on a large number of patients with primary metastatic osteosarcoma, we further proved that older age (≥25 years) was associated with poor survival in osteosarcoma patients. As previously reported, the primary osteosarcoma in elderly patients showed different characteristics from younger patients, such as more involvement on axial bone and low response to chemotherapy.^29^ Therefore, age should be one of the most important factors considered for personalized treatment. Age showed no significant correlation with the survival of patients with bone metastasis.

In the present study, surgery on the primary site was found to be significantly associated with an increased lifespan of patients with lung metastasis. The benefit of surgery has been widely reported in many previous studies, and surgery is accepted as a fundamental strategy to improve OS. Thus, to improve the survival of patients, aggressive surgery on the primary lesion was recommended. Resection of the isolated lung metastasis was also reported to be associated with improved survival.^12^, ^21^ In contrast, surgery did not significantly improve survival in patients with bone metastasis in the present study. Due to the unrecorded information on surgery type by the SEER database, we could not perform further investigation on the effect of surgery on survival. To determine the correlation between surgery and the survival of osteosarcoma patients with distant metastasis, further cohorts with detailed information on surgical treatment are needed.

### Limitations

Although our study identified the largest sample of distant metastasis in primary osteosarcoma, the results should be interpreted with caution. The SEER database does not record asymptomatic cases with distant metastasis, which may have led to an underestimation of the real occurrence rate of distant metastasis in osteosarcoma patients. The sample size was not large enough to perform further analysis to reveal the prognostic factors in patients with liver or brain metastasis. In addition, one of the key factors in the correlation between age and survival is chemotherapy. However, the SEER database does not record detailed information on chemotherapy. Thus, further analysis of the correlation between chemotherapy and survival could not be performed. All weaknesses in the SEER database need to be investigated in the future with available data.

### Conclusion

In summary, approximately 18.9% of osteosarcoma patients were initially diagnosed with distant metastasis, and the lung was the most common metastatic site. More distant metastases were noticed in patients younger than 25 years or older than 60 years, with T2 and T3 stage, and with N1 stage in the entire cohort. Patients older than 25 years showed poor survival. Surgery at the primary site was a protective factor for the survival of patients with lung metastasis.

## Supporting information


**Supplementary Table S1** Description of the SEER osteosarcoma patients with distant metastasis at diagnosis

**Supplementary Table S2** Univariate logistic regression analyzing the risk factors for developing distant metastases in patients diagnosed with osteosarcoma (diagnosed between January 2010 and December 2015)

**Supplementary Table S3** Multivariate logistic regression analyzing the risk factors for development of distant metastasis in osteosarcoma patients diagnosed between 2010 and 2015

**Supplementary Table S4** Overall survival of osteosarcoma patients with or without distant metastases (between January 2010 and December 2014)

**Supplementary Table S5** Univariate Cox regression analyzing the prognostic factors for osteosarcoma patients with distant metastases (between January 2010 and December 2014)

**Supplementary Table S6** Multivariate Cox regression analyzing the prognostic factors of osteosarcoma patients with distant metastases (diagnosed between 2010 and 2014)
