# Supplementary material for: Risk and Prognostic Factors for Different Organ Metastasis in Primary Osteosarcoma: A Large Population‐Based Analysis
Source: Orthop Surg. 2022 Mar 16;14(4):714–9. doi: 10.1111/os.13243 (PMC9002071; doi:10.1111/os.13243)
Supplement: Supplementary file 1 — Supplementary Table S1 Description of the SEER osteosarcoma patients with distant metastasis at diagnosis [file OS-14-714-s004.docx]

Supplementary table S1. Description of the SEER osteosarcoma patients with distant metastasis at diagnosis.

| **Subject characteristics** | **Total-Met** | | | | **Bone-Met** | | | | **Brain-Met** | | | | **Liver-Met** | | | | **Lung-Met** | | | |
| --- | --- | --- | --- | --- | --- | --- | --- | --- | --- | --- | --- | --- | --- | --- | --- | --- | --- | --- | --- | --- |
|  | **No**  **N (%)** | **Yes**  **N (%)** | **χ2** | ***P*** | **No**  **N (%)** | **Yes**  **N (%)** | **χ2** | ***P*** | **No**  **N (%)** | **Yes**  **N (%)** | **χ2** | ***P*** | **No**  **N (%)** | **Yes**  **N (%)** | **χ2** | ***P*** | **No**  **N (%)** | **Yes**  **N (%)** | **χ2** | ***P*** |
| **Sex** |  |  | 6.732 | 0.009 |  |  | 0.016 | 0.896 |  |  | 1.967 | 0.161 |  |  | 0.078 | 0.780 |  |  | 5.331 | 0.021 |
| Male | 635(78.69) | 172(21.31) |  |  | 774(95.91) | 33(4.09) |  |  | 802(99.38) | 5(0.62) |  |  | 803(99.50) | 4(0.50) |  |  | 663(82.16) | 144(17.84) |  |  |
| Female | 557(84.01) | 106(15.99) |  |  | 635(95.78) | 28(4.22) |  |  | 662(99.85) | 1(0.15) |  |  | 659(99.40) | 4(0.60) |  |  | 574(86.58) | 89(13.42) |  |  |
| **Age** |  |  | 13.357 | 0.001 |  |  | 4.668 | 0.097 |  |  | 1.609 | 0.447 |  |  | 13.223 | 0.001 |  |  | 8.990 | 0.011 |
| 0-24 | 678(80.43) | 165(19.57) |  |  | 809(95.97) | 34(4.03) |  |  | 841(99.76) | 2(0.24) |  |  | 843(100.00) | 0(0.00) |  |  | 701(83.16) | 142(16.84) |  |  |
| 25-59 | 360(85.92) | 59(14.08) |  |  | 406(96.90) | 13(3.10) |  |  | 416(99.28) | 3(0.72) |  |  | 415(99.05) | 4(0.95) |  |  | 370(88.31) | 49(11.69) |  |  |
| ≥60 | 154(74.04) | 54(25.96) |  |  | 194(93.27) | 14(6.73) |  |  | 207(99.52) | 1(0.48) |  |  | 204(98.08) | 4(1.92) |  |  | 166(79.81) | 42(20.19) |  |  |
| **Race** |  |  | 0.428 | 0.934 |  |  | 2.000 | 0.572 |  |  | 1.816 | 0.612 |  |  | 0.211 | 0.976 |  |  | 0.101 | 0.992 |
| White | 899(81.36) | 206(18.64) |  |  | 1,061(96.02) | 44(3.98) |  |  | 1,101(99.64) | 4(0.36) |  |  | 1,099(99.46) | 6(0.54) |  |  | 931(84.25) | 174(15.75) |  |  |
| Black | 183(79.57) | 47(20.43) |  |  | 217(94.35) | 13(5.65) |  |  | 228(99.13) | 2(0.87) |  |  | 229(99.57) | 1(0.43) |  |  | 192(83.48) | 38(16.52) |  |  |
| Others | 105(81.40) | 24(18.60) |  |  | 125(96.90) | 4(3.10) |  |  | 129(100.00) | 0(0.00) |  |  | 128(99.22) | 1(0.78) |  |  | 109(84.50) | 20(15.50) |  |  |
| Unknown | 5(83.33) | 1(16.67) |  |  | 6(100.00) | 0(0.00) |  |  | 6(100.00) | 0(0.00) |  |  | 6(100.00) | 0(0.00) |  |  | 5(83.33) | 1(16.67) |  |  |
| **Insurance recode** |  |  | 1.134 | 0.567 |  |  | 0.643 | 0.725 |  |  | 0.333 | 0.847 |  |  | 0.445 | 0.801 |  |  | 1.737 | 0.420 |
| Uninsured | 42(85.71) | 7(14.29) |  |  | 47(95.92) | 2(4.08) |  |  | 49(100.00) | 0(0.00) |  |  | 49(100.00) | 0(0.00) |  |  | 42(85.71) | 7(14.29) |  |  |
| Insured | 1,126(80.83) | 267(19.17) |  |  | 1,336(95.91) | 57(4.09) |  |  | 1,387(99.57) | 6(0.43) |  |  | 1,385(99.43) | 8(0.57) |  |  | 1,169(83.92) | 224(16.08) |  |  |
| Unknown | 24(85.71) | 4(14.29) |  |  | 26(92.86) | 2(7.14) |  |  | 28(100.00) | 0(0.00) |  |  | 28(100.00) | 0(0.00) |  |  | 26(92.86) | 2(7.14) |  |  |
| **Marital status** |  |  | 2.408 | 0.300 |  |  | 1.092 | 0.579 |  |  | 2.093 | 0.351 |  |  | 6.791 | 0.034 |  |  | 3.181 | 0.204 |
| Unmarried | 882(80.84) | 209(19.16) |  |  | 1,049(96.15) | 42(3.85) |  |  | 1,085(99.45) | 6(0.55) |  |  | 1,088(99.73) | 3(0.27) |  |  | 914(83.78) | 177(16.22) |  |  |
| Married | 287(82.71) | 60(17.29) |  |  | 330(95.10) | 17(4.90) |  |  | 347(100.00) | 0(0.00) |  |  | 342(98.56) | 5(1.44) |  |  | 299(86.17) | 48(13.83) |  |  |
| Unknown | 23(71.88) | 9(28.13) |  |  | 30(93.75) | 2(6.25) |  |  | 32(100.00) | 0(0.00) |  |  | 32(100.00) | 0(0.00) |  |  | 24(75.00) | 8(25.00) |  |  |
| **Primary site** |  |  | 17.256 | <0.001 |  |  | 15.809 | <0.001 |  |  | 1.779 | 0.411 |  |  | 80.088 | <0.001 |  |  | 6.990 | 0.030 |
| Extremity | 866(81.47) | 197(18.53) |  |  | 1,031(96.99) | 32(3.01) |  |  | 1,060(99.72) | 3(0.28) |  |  | 1,061(99.81) | 2(0.19) |  |  | 890(83.73) | 173(16.27) |  |  |
| Axial | 317(81.91) | 70(18.09) |  |  | 361(93.28) | 26(6.72) |  |  | 384(99.22) | 3(0.78) |  |  | 384(99.22) | 3(0.78) |  |  | 334(86.30) | 53(13.70) |  |  |
| Unknown | 9(45.00) | 11(55.00) |  |  | 17(85.00) | 3(15.00) |  |  | 20(100.00) | 0(0.00) |  |  | 17(85.00) | 3(15.00) |  |  | 13(65.00) | 7(35.00) |  |  |
| **Year of diagnosis** |  |  | 2.492 | 0.778 |  |  | 0.988 | 0.964 |  |  | 6.159 | 0.291 |  |  | 4.583 | 0.469 |  |  | 3.913 | 0.562 |
| 2010 | 185(82.96) | 38(17.04) |  |  | 212(95.07) | 11(4.93) |  |  | 223(100.00) | 0(0.00) |  |  | 223(100.00) | 0(0.00) |  |  | 189(84.75) | 34(15.25) |  |  |
| 2011 | 201(80.72) | 48(19.28) |  |  | 238(95.58) | 11(4.42) |  |  | 249(100.00) | 0(0.00) |  |  | 248(99.60) | 1(0.40) |  |  | 214(85.94) | 35(14.06) |  |  |
| 2012 | 195(78.00) | 55(22.00) |  |  | 239(95.60) | 11(4.40) |  |  | 248(99.20) | 2(0.80) |  |  | 249(99.60) | 1(0.40) |  |  | 201(80.40) | 49(19.60) |  |  |
| 2013 | 175(80.65) | 42(19.35) |  |  | 210(96.77) | 7(3.23) |  |  | 215(99.08) | 2(0.92) |  |  | 214(98.62) | 3(1.38) |  |  | 184(84.79) | 33(15.21) |  |  |
| 2014 | 217(81.89) | 48(18.11) |  |  | 255(96.23) | 10(3.77) |  |  | 263(99.25) | 2(0.75) |  |  | 263(99.25) | 2(0.75) |  |  | 227(85.66) | 38(14.34) |  |  |
| 2015 | 219(82.33) | 47(17.67) |  |  | 255(95.86) | 11(4.14) |  |  | 266(100.00) | 0(0.00) |  |  | 265(99.62) | 1(0.38) |  |  | 222(83.46) | 44(16.54) |  |  |
| **Histology** |  |  | 21.732 | 0.001 |  |  | 7.522 | 0.275 |  |  | 3.040 | 0.804 |  |  | 6.084 | 0.414 |  |  | 14.785 | 0.022 |
| Osteosarcoma, NOS | 768(78.61) | 209(21.39) |  |  | 933(95.50) | 44(4.50) |  |  | 971(99.39) | 6(0.61) |  |  | 970(99.28) | 7(0.72) |  |  | 804(82.29) | 173(17.71) |  |  |
| Chondroblastic | 181(81.90) | 40(18.10) |  |  | 213(96.38) | 8(3.62) |  |  | 221(100.00) | 0(0.00) |  |  | 221(100.00) | 0(0.00) |  |  | 188(85.07) | 33(14.93) |  |  |
| Central | 68(88.31) | 9(11.69) |  |  | 76(98.70) | 1(1.30) |  |  | 77(100.00) | 0(0.00) |  |  | 77(100.00) | 0(0.00) |  |  | 69(89.61) | 8(10.39) |  |  |
| Parosteal | 67(97.10) | 2(2.90) |  |  | 68(98.55) | 1(1.45) |  |  | 69(100.00) | 0(0.00) |  |  | 69(100.00) | 0(0.00) |  |  | 67(97.10) | 2(2.90) |  |  |
| Fibroblastic | 40(86.96) | 6(13.04) |  |  | 44(95.65) | 2(4.35) |  |  | 46(100.00) | 0(0.00) |  |  | 46(100.00) | 0(0.00) |  |  | 40(86.96) | 6(13.04) |  |  |
| Telangiectatic | 38(90.48) | 4(9.52) |  |  | 41(97.62) | 1(2.38) |  |  | 42(100.00) | 0(0.00) |  |  | 42(100.00) | 0(0.00) |  |  | 38(90.48) | 4(9.52) |  |  |
| Others | 30(78.95) | 8(21.05) |  |  | 34(89.47) | 4(10.53) |  |  | 38(100.00) | 0(0.00) |  |  | 37(97.37) | 1(2.63) |  |  | 31(81.58) | 7(18.42) |  |  |
| **Grade** |  |  | 25.992 | <0.001 |  |  | 10.989 | 0.027 |  |  | 1.173 | 0.883 |  |  | 3.825 | 0.430 |  |  | 19.202 | 0.001 |
| Grade I | 58(98.31) | 1(1.69) |  |  | 59(100.00) | 0(0.00) |  |  | 59(100.00) | 0(0.00) |  |  | 59(100.00) | 0(0.00) |  |  | 58(98.31) | 1(1.69) |  |  |
| Grade II | 81(95.29) | 4(4.71) |  |  | 85(100.00) | 0(0.00) |  |  | 85(100.00) | 0(0.00) |  |  | 85(100.00) | 0(0.00) |  |  | 81(95.29) | 4(4.71) |  |  |
| Grade III | 279(78.15) | 78(21.85) |  |  | 347(97.20) | 10(2.80) |  |  | 355(99.44) | 2(0.56) |  |  | 355(99.44) | 2(0.56) |  |  | 291(81.51) | 66(18.49) |  |  |
| Grade IV | 504(80.51) | 122(19.49) |  |  | 594(94.89) | 32(5.11) |  |  | 624(99.68) | 2(0.32) |  |  | 624(99.68) | 2(0.32) |  |  | 522(83.39) | 104(16.61) |  |  |
| Unknown | 270(78.72) | 73(21.28) |  |  | 324(94.46) | 19(5.54) |  |  | 341(99.42) | 2(0.58) |  |  | 339(98.83) | 4(1.17) |  |  | 285(83.09) | 58(16.91) |  |  |
| **T stage** |  |  | 69.294 | <0.001 |  |  | 44.521 | <0.001 |  |  | 0.252 | 0.969 |  |  | 40.632 | <0.001 |  |  | 65.065 | <0.001 |
| T1 | 491(89.93) | 55(10.07) |  |  | 534(97.80) | 12(2.20) |  |  | 544(99.63) | 2(0.37) |  |  | 546(100.00) | 0(0.00) |  |  | 506(92.67) | 40(7.33) |  |  |
| T2 | 556(79.32) | 145(20.68) |  |  | 676(96.43) | 25(3.57) |  |  | 698(99.57) | 3(0.43) |  |  | 700(99.86) | 1(0.14) |  |  | 571(81.46) | 130(18.54) |  |  |
| T3 | 20(55.56) | 16(44.44) |  |  | 28(77.78) | 8(22.22) |  |  | 36(100.00) | 0(0.00) |  |  | 36(100.00) | 0(0.00) |  |  | 21(58.33) | 15(41.67) |  |  |
| Unknown | 125(66.84) | 62(33.16) |  |  | 171(91.44) | 16(8.56) |  |  | 186(99.47) | 1(0.53) |  |  | 180(96.26) | 7(3.74) |  |  | 139(74.33) | 48(25.67) |  |  |
| **N stage** |  |  | 62.046 | <0.001 |  |  | 22.764 | <0.001 |  |  | 28.558 | <0.001 |  |  | 15.192 | 0.001 |  |  | 46.181 | <0.001 |
| N0 | 1,151(83.04) | 235(16.96) |  |  | 1,336(96.39) | 50(3.61) |  |  | 1.382(99.71) | 4(0.29) |  |  | 1,381(99.64) | 5(0.36) |  |  | 1,188(85.71) | 198(14.29) |  |  |
| N1 | 13(41.94) | 18(58.06) |  |  | 25(80.65) | 6(19.35) |  |  | 29(93.55) | 2(6.45) |  |  | 30(96.77) | 1(3.23) |  |  | 16(51.61) | 15(48.39) |  |  |
| Unknown | 28(52.83) | 25(47.17) |  |  | 48(90.57) | 5(9.43) |  |  | 53(100.00) | 0(0.00) |  |  | 51(96.23) | 2(3.77) |  |  | 33(62.26) | 20(37.74) |  |  |
| **Number of Met** |  |  | 203.609 | <0.001 |  |  | 8.792 | 0.040 |  |  | 4.279 | 0.039 |  |  | 2.597 | 0.107 |  |  | 3.507 | 0.061 |
| ≤1 | 1,192(83.71) | 232(16.29) |  |  | 1,403(95.96) | 59(4.04) |  |  | 1,424(99.65) | 5(0.35) |  |  | 1,420(99.51) | 7(0.49) |  |  | 1,235(84.24) | 231(15.76) |  |  |
| ＞1 | 0(0.00) | 46(100.00) |  |  | 6(75.00) | 2(25.00) |  |  | 40(97.56) | 1(2.44) |  |  | 42(97.67) | 1(2.33) |  |  | 2(50.00) | 2(50.00) |  |  |
| **Vital status** |  |  | 145.664 | <0.001 |  |  | 47.995 | <0.001 |  |  | 14.523 | <0.001 |  |  | 8.100 | 0.004 |  |  | 90.635 | <0.001 |
| Alive | 925(89.03) | 114(10.97) |  |  | 1,020(98.17) | 19(1.83) |  |  | 1,039(100.00) | 0(0.00) |  |  | 1,037(99.81) | 2(0.19) |  |  | 935(89.99) | 104(10.01) |  |  |
| Dead | 267(61.95) | 164(38.05) |  |  | 389(90.26) | 42(9.74) |  |  | 425(98.61) | 6(1.39) |  |  | 425(98.61) | 6(1.39) |  |  | 302(70.07) | 129(29.93) |  |  |
| **Surg (prim)** |  |  | 131.269 | <0.001 |  |  | 56.716 | <0.001 |  |  | 20.125 | <0.001 |  |  | 30.305 | <0.001 |  |  | 92.590 | <0.001 |
| None | 129(54.43) | 108(45.57) |  |  | 206(86.92) | 31(13.08) |  |  | 232(97.89) | 5(2.11) |  |  | 230(97.05) | 7(2.95) |  |  | 150(63.29) | 87(36.71) |  |  |
| Yes | 1,060(86.25) | 169(13.75) |  |  | 1,199(97.56) | 30(2.44) |  |  | 1,228(99.92) | 1(0.08) |  |  | 1,228(99.92) | 1(0.08) |  |  | 1,083(88.12) | 146(11.88) |  |  |
| Unknown | 3(75.00) | 1(25.00) |  |  | 4(100.00) | 0(0.00) |  |  | 4(100.00) | 0(0.00) |  |  | 4(100.00) | 0(0.00) |  |  | 4(100.00) | 0(0.00) |  |  |

Abbreviations: SEER: Surveillance, Epidemiology, and End Result; Met: Metastasis; Surg (prim): surgery of primary tumor.
