# Supplementary material for: Risk and Prognostic Factors for Different Organ Metastasis in Primary Osteosarcoma: A Large Population‐Based Analysis
Source: Orthop Surg. 2022 Mar 16;14(4):714–9. doi: 10.1111/os.13243 (PMC9002071; doi:10.1111/os.13243)
Supplement: Supplementary file 2 — Supplementary Table S2 Univariate logistic regression analyzing the risk factors for developing distant metastases in patients diagnosed with osteosarcoma (diagnosed between January 2010 and December 2015) [file OS-14-714-s001.docx]

Supplementary table S2. Univariate Logistic Regression analyzing the risk factors for developing distant metastases in patients diagnosed with osteosarcoma (Diagnosed between January 2010 and December 2015).

| **Subject characteristics** | **Total-Met** | | **Bone-Met** | | **Brain-Met** | | **Liver-Met** | | **Lung-Met** | |
| --- | --- | --- | --- | --- | --- | --- | --- | --- | --- | --- |
|  | **OR (95%CI)** | ***P-value*** | **OR (95%CI)** | ***P-value*** | **OR (95%CI)** | ***P-value*** | **OR (95%CI)** | ***P-value*** | **OR (95%CI)** | ***P-value*** |
| **Sex** |  |  |  |  |  |  |  |  |  |  |
| Male | 1.00 (Reference) |  | 1.00 (Reference) |  | 1.00 (Reference) |  | 1.00 (Reference) |  | 1.00 (Reference) |  |
| Female | 0.70 (0.54-0.92) | 0.010 | 1.03 (0.62-1.73) | 0.898 | 0.24 (0.03-2.08) | 0.196 | 1.22 (0.30-4.89) | 0.780 | 0.71 (0.54-0.95) | 0.021 |
| **Age** |  |  |  |  |  |  |  |  |  |  |
| 0-24 | 1.00 (Reference) |  | 1.00 (Reference) |  | 1.00 (Reference) |  | 1.00 (Reference) |  | 1.00 (Reference) |  |
| 25-59 | 0.67 (0.49-0.93) | 0.017 | 0.76 (0.40-1.46) | 0.412 | 3.03 (0.50-18.22) | 0.225 | NA | NA | 0.65 (0.46-0.93) | 0.017 |
| ≥60 | 1.44 (1.01-2.05) | 0.043 | 1.72 (0.90-3.26) | 0.099 | 2.03 (0.18-22.51) | 0.564 | NA | NA | 1.25 (0.85-1.83) | 0.256 |
| **Race** |  |  |  |  |  |  |  |  |  |  |
| White | 1.00 (Reference) |  | 1.00 (Reference) |  | 1.00 (Reference) |  | 1.00 (Reference) |  | 1.00 (Reference) |  |
| Black | 1.12 (0.79-1.60) | 0.528 | 1.44 (0.76-2.73) | 0.257 | 2.41 (0.44-13.26) | 0.310 | 0.80 (0.10-6.68) | 0.837 | 1.06 (0.72-1.55) | 0.770 |
| Others | 1.00 (0.62-1.59) | 0.992 | 0.77 (0.26-2.18) | 0.625 | NA | NA | 1.43 (0.17-11.98) | 0.741 | 0.98 (0.59-1.62) | 0.943 |
| Unknown | NA | NA | NA | NA | NA | NA | NA | NA | NA | NA |
| **Insurance recode** |  |  |  |  |  |  |  |  |  |  |
| Uninsured | 1.00 (Reference) |  | 1.00 (Reference) |  | NA | NA | NA | NA | 1.00 (Reference) |  |
| Insured | 1.42 (0.63-3.20) | 0.394 | 1.00 (0.24-4.23) | 0.997 | NA | NA | NA | NA | 1.15 (0.51-2.59) | 0.737 |
| Unknown | NA | NA | NA | NA | NA | NA | NA | NA | NA | NA |
| **Marital status** |  |  |  |  |  |  |  |  |  |  |
| Unmarried | 1.00 (Reference) |  | 1.00 (Reference) |  | NA | NA | 1.00 (Reference) |  | 1.00 (Reference) |  |
| Married | 0.88 (0.64-1.21) | 0.438 | 1.29 (0.72-2.29) | 0.392 | NA | NA | 5.30 (1.26-22.30) | 0.023 | 0.83 (0.59-1.17) | 0.286 |
| Unknown | NA | NA | NA | NA | NA | NA | NA | NA | NA | NA |
| **Primary site** |  |  |  |  |  |  |  |  |  |  |
| Extremity | 1.00 (Reference) |  | 1.00 (Reference) |  | 1.00 (Reference) |  | 1.00 (Reference) |  | 1.00 (Reference) |  |
| Axial | 0.97 (0.72-1.31) | 0.847 | 2.32 (1.36-3.95) | 0.002 | 2.76 (0.55-13.73) | 0.215 | 4.14 (0.69-24.90) | 0.120 | 0.82 (0.59-1.14) | 0.232 |
| Unknown | NA | NA | NA | NA | NA | NA | NA | NA | NA | NA |
| **Histology** |  |  |  |  |  |  |  |  |  |  |
| Osteosarcoma, NOS | 1.00 (Reference) |  | 1.00 (Reference) |  | NA | NA | 1.00 (Reference) |  | 1.00 (Reference) |  |
| Chondroblastic | 0.81 (0.56-1.18) | 0.277 | 0.80 (0.37-1.72) | 0.561 | NA | NA | NA | NA | 0.82 (0.54-1.22) | 0.324 |
| Central | 0.49 (0.24-0.99) | 0.047 | 0.28 (0.04-2.05) | 0.210 | NA | NA | NA | NA | 0.54 (0.55-1.14) | 0.106 |
| Parosteal | 0.11 (0.03-0.45) | 0.002 | 0.31 (0.04-2.30) | 0.253 | NA | NA | NA | NA | 0.14 (0.53-0.57) | 0.006 |
| Fibroblastic | 0.55 (0.23-1.32) | 0.180 | 0.96 (0.23-4.10) | 0.960 | NA | NA | NA | NA | 0.70 (0.59-1.67) | 0.418 |
| Telangiectatic | 0.39 (0.14-1.10) | 0.074 | 0.52 (0.07-3.85) | 0.520 | NA | NA | NA | NA | 0.49 (0.57-1.39) | 0.179 |
| Others | 0.98 (0.44-2.17) | 0.960 | 2.49 (0.85-7.34) | 0.097 | NA | NA | 3.75 (0.45-31.23) | 0.222 | 1.05 (0.55-2.42) | 0.910 |
| **Grade** |  |  |  |  |  |  |  |  |  |  |
| Grade I | 1.00 (Reference) |  | NA | NA | NA | NA | NA | NA | 1.00 (Reference) |  |
| Grade II | 2.86 (0.31-26.29) | 0.352 | NA | NA | NA | NA | NA | NA | 2.86 (2.71-26.29) | 0.352 |
| Grade III | 16.22 (2.21-118.94) | 0.006 | NA | NA | NA | NA | NA | NA | 13.15 (1.79-96.69) | 0.011 |
| Grade IV | 14.04 (1.93-102.36) | 0.009 | NA | NA | NA | NA | NA | NA | 11.56 (1.58-84.36) | 0.016 |
| Unknown | NA | NA | NA | NA | NA | NA | NA | NA | NA | NA |
| **T stage** |  |  |  |  |  |  |  |  |  |  |
| T1 | 1.00 (Reference) |  | 1.00 (Reference) |  | 1.00 (Reference) |  | NA | NA | 1.00 (Reference) |  |
| T2 | 2.33 (1.67-3.25) | <0.001 | 1.65 (0.82-3.31) | 0.162 | 1.17 (0.19-7.02) | 0.864 | NA | NA | 2.88 (1.98-4.19) | <0.001 |
| T3 | 7.14 (3.50-14.58) | <0.001 | 12.71 (4.81-33.61) | <0.001 | NA | NA | NA | NA | 9.04 (4.33-18.87) | <0.001 |
| Unknown | NA | NA | NA | NA | NA | NA | NA | NA | NA | NA |
| **N stage** |  |  |  |  |  |  |  |  |  |  |
| N0 | 1.00 (Reference) |  | 1.00 (Reference) |  | 1.00 (Reference) |  | 1.00 (Reference) |  | 1.00 (Reference) |  |
| N1 | 6.78(3.28-14.03) | <0.001 | 6.41(2.52-16.33) | <0.001 | 23.83(4.20-135.31) | <0.001 | 9.21(1.04-81.22) | 0.046 | 5.62(2.74-11.56) | <0.001 |
| Unknown | NA | NA | NA | NA | NA | NA | NA | NA | NA | NA |
| **Number of metastasis** |  |  |  |  |  |  |  |  |  |  |
| ≤1 | NA | NA | 1.00 (Reference) |  | 1.00 (Reference) |  | 1.00 (Reference) |  | NA | NA |
| ＞1 | NA | NA | 7.93 (1.57-40.11) | 0.012 | 7.12 (0.81-62.35) | 0.076 | 4.83 (0.58-40.14) | 0.145 | NA | NA |

Abbreviations: Met: Metastases; OR: odds ratio.
