# Supplementary material for: Risk and Prognostic Factors for Different Organ Metastasis in Primary Osteosarcoma: A Large Population‐Based Analysis
Source: Orthop Surg. 2022 Mar 16;14(4):714–9. doi: 10.1111/os.13243 (PMC9002071; doi:10.1111/os.13243)
Supplement: Supplementary file 3 — Supplementary Table S3 Multivariate logistic regression analyzing the risk factors for development of distant metastasis in osteosarcoma patients diagnosed between 2010 and 2015 [file OS-14-714-s002.docx]

Supplementary table S3. Multivariate Logistic Regression analyzing the risk factors for development of distant metastasis in osteosarcoma patients diagnosed between 2010 and 2015.

| **Subject characteristics** | **Total-Met** | | **Bone-Met** | | **Brain-Met** | | **Liver-Met** | | **Lung-Met** | |
| --- | --- | --- | --- | --- | --- | --- | --- | --- | --- | --- |
|  | **OR (95%CI)** | ***P-value*** | **OR (95%CI)** | ***P-value*** | **OR (95%CI)** | ***P-value*** | **OR (95%CI)** | ***P-value*** | **OR (95%CI)** | ***P-value*** |
| **Sex** |  |  |  |  |  |  |  |  |  |  |
| Male | 1.00 (Reference) |  | 1.00 (Reference) |  | 1.00 (Reference) |  | 1.00 (Reference) |  | 1.00 (Reference) |  |
| Female | 0.89(0.62-1.28) | 0.545 | 1.37(0.73-2.57) | 0.325 | 0.23(0.02-2.29) | 0.208 | 3.01(0.52-17.51) | 0.220 | 0.86(0.58-1.27) | 0.453 |
| **Age** |  |  |  |  |  |  |  |  |  |  |
| 0-24 | 1.00 (Reference) |  | 1.00 (Reference) |  | 1.00 (Reference) |  | NA | NA | 1.00 (Reference) |  |
| 25-59 | 0.59(0.37-0.95) | 0.028 | 0.49(0.21-1.12) | 0.091 | 2.84(0.36-22.45) | 0.323 | NA | NA | 0.58(0.35-0.96) | 0.034 |
| ≥60 | 1.06(0.62-1.80) | 0.845 | 0.74(0.29-1.92) | 0.536 | 3.27(0.25-43.44) | 0.369 | NA | NA | 0.88(0.48-1.61) | 0.672 |
| **Marital status** |  |  |  |  |  |  |  |  |  |  |
| Unmarried | NA | NA | NA | NA | NA | NA | 1.00 (Reference) |  | NA | NA |
| Married | NA | NA | NA | NA | NA | NA | 3.61(0.70-18.53) | 0.124 | NA | NA |
| Unknown | NA | NA | NA | NA | NA | NA | NA | NA | NA | NA |
| **Primary site** |  |  |  |  |  |  |  |  |  |  |
| Extremity | NA | NA | 1.00 (Reference) |  | NA | NA | NA | NA | NA | NA |
| Axial | NA | NA | 2.77(1.34-5.73) | 0.006 | NA | NA | NA | NA | NA | NA |
| Unknown | NA | NA | NA | NA | NA | NA | NA | NA | NA | NA |
| **Histology** |  |  |  |  |  |  |  |  |  |  |
| Osteosarcoma, NOS | 1.00 (Reference) |  | NA | NA | NA | NA | NA | NA | 1.00 (Reference) |  |
| Chondroblastic | 0.74(0.45-1.21) | 0.230 | NA | NA | NA | NA | NA | NA | 0.64(0.37-1.12) | 0.120 |
| Central | 0.65(0.28-1.52) | 0.317 | NA | NA | NA | NA | NA | NA | 0.67(0.27-1.65) | 0.383 |
| Parosteal | 0.56(0.12-2.73) | 0.473 | NA | NA | NA | NA | NA | NA | 0.64(0.13-3.23) | 0.593 |
| Fibroblastic | 0.61(0.18-2.10) | 0.435 | NA | NA | NA | NA | NA | NA | 0.77(0.22-2.67) | 0.684 |
| Telangiectatic | 0.30(0.07-1.34) | 0.115 | NA | NA | NA | NA | NA | NA | 0.37(0.08-1.69) | 0.201 |
| Others | 0.99(0.32-6.10) | 0.993 | NA | NA | NA | NA | NA | NA | 1.23(0.39-3.86) | 0.723 |
| **Grade** |  |  |  |  |  |  |  |  |  |  |
| Grade I | NA | NA | NA | NA | NA | NA | NA | NA | 1.00 (Reference) |  |
| Grade II | NA | NA | NA | NA | NA | NA | NA | NA | 1.99(0.20-20.05) | 0.560 |
| Grade III | NA | NA | NA | NA | NA | NA | NA | NA | 5.69(0.70-46.49) | 0.105 |
| Grade IV | NA | NA | NA | NA | NA | NA | NA | NA | 5.00(0.62-40.37) | 0.131 |
| Unknown | NA | NA | NA | NA | NA | NA | NA | NA | NA | NA |
| **T stage** |  |  |  |  |  |  |  |  |  |  |
| T1 | 1.00 (Reference) |  | 1.00 (Reference) |  | 1.00 (Reference) |  | NA | NA | 1.00 (Reference) |  |
| T2 | 1.91(1.28-2.84) | 0.001 | 2.03(0.97-4.25) | 0.060 | 1.06(0.16-6.88) | 0.951 | NA | NA | 2.46(1.57-3.86) | <0.001 |
| T3 | 4.48(1.78-11.30) | 0.001 | 14.64(5.07-42.26) | <0.001 | NA | NA | NA | NA | 5.54(2.11-14.53) | <0.001 |
| Unknown | NA | NA | NA | NA | NA | NA | NA | NA | NA | NA |
| **N stage** |  |  |  |  |  |  |  |  |  |  |
| N0 | 1.00 (Reference) |  | 1.00 (Reference) |  | 1.00 (Reference) |  | 1.00 (Reference) |  | 1.00 (Reference) |  |
| N1 | 6.66(2.68-16.56) | <0.001 | 9.09(3.32-24.90) | <0.001 | 5.39(0.49-59.51) | 0.169 | 13.98(1.44-135.98) | 0.023 | 5.81(2.34-14.45) | <0.001 |
| Unknown | NA | NA | NA | NA | NA | NA | NA | NA | NA | NA |
| **Number of metastasis** |  |  |  |  |  |  |  |  |  |  |
| ≤1 | NA | NA | 1.00 (Reference) |  | 1.00 (Reference) |  | NA | NA | NA | NA |
| ＞1 | NA | NA | 7.02(0.65-76.16) | 0.109 | 16.02(1.17-219.19) | 0.038 | NA | NA | NA | NA |

Abbreviations: Met: Metastasis; OR: odds ratio.
