# Supplementary material for: Risk and Prognostic Factors for Different Organ Metastasis in Primary Osteosarcoma: A Large Population‐Based Analysis
Source: Orthop Surg. 2022 Mar 16;14(4):714–9. doi: 10.1111/os.13243 (PMC9002071; doi:10.1111/os.13243)
Supplement: Supplementary file 4 — Supplementary Table S4 Overall survival of osteosarcoma patients with or without distant metastases (between January 2010 and December 2014) [file OS-14-714-s005.docx]

Supplementary table S4. Overall survival of osteosarcoma patients with or without distant metastases (between January 2010 and December 2014).

| **Subject**  **characteristics** |  | **Log-rank Test** | |
| --- | --- | --- | --- |
|  |  | **χ2** | ***P-value*** |
| **Sex** |  |  |  |
| Male |  | 152.1 | *<0.001* |
| Female |  | 89.4 | *<0.001* |
| **Age** |  |  |  |
| 0-24 |  | 137.2 | *<0.001* |
| 25-59 |  | 77.5 | *<0.001* |
| ≥60 |  | 57.9 | *<0.001* |
| **Race** |  |  |  |
| White |  | 214.1 | *<0.001* |
| Black |  | 22.1 | *<0.001* |
| Others |  | 18.4 | *<0.001* |
| **Insurance recode** |  |  |  |
| Uninsured |  | 5.5 | *0.019* |
| Insured |  | 239.1 | *<0.001* |
| **Marital status** |  |  |  |
| Unmarried |  | 174.8 | *<0.001* |
| Married |  | 98.2 | *<0.001* |
| **Primary site** |  |  |  |
| Extremity |  | 179.9 | *<0.001* |
| Axial |  | 89.0 | *<0.001* |
| **Histology** |  |  |  |
| Osteosarcoma, NOS |  | 188.7 | *<0.001* |
| Chondroblastic |  | 10.8 | *0.001* |
| Central |  | 2.8 | *0.094* |
| Parosteal |  | 8.4 | *0.004* |
| Fibroblastic |  | 3.3 | *0.070* |
| Telangiectatic |  | 0.3 | *0.567* |
| Others |  | 40.0 | *<0.001* |
| **Grade** |  |  |  |
| Grade I |  | - | *-* |
| Grade II |  | 42.9 | *<0.001* |
| Grade III |  | 57.0 | *<0.001* |
| Grade IV |  | 81.8 | *<0.001* |
| **T stage** |  |  |  |
| T1 |  | 46.4 | *<0.001* |
| T2 |  | 112.3 | *<0.001* |
| T3 |  | 5.3 | *0.021* |
| **N stage** |  |  |  |
| N0 |  | 215.9 | *<0.001* |
| N1 |  | 3.5 | *0.062* |
| **Number of mets** |  |  |  |
| ≤1 |  | 194.9 | *<0.001* |
| >1 |  | - | *-* |
| **Surg (prim)** |  |  |  |
| None |  | 32.6 | *<0.001* |
| Yes |  | 106.1 | *<0.001* |

Abbreviations: Met=Metastases.
