# Supplementary material for: Risk and Prognostic Factors for Different Organ Metastasis in Primary Osteosarcoma: A Large Population‐Based Analysis
Source: Orthop Surg. 2022 Mar 16;14(4):714–9. doi: 10.1111/os.13243 (PMC9002071; doi:10.1111/os.13243)
Supplement: Supplementary file 5 — Supplementary Table S5 Univariate Cox regression analyzing the prognostic factors for osteosarcoma patients with distant metastases (between January 2010 and December 2014) [file OS-14-714-s006.docx]

Supplementary table S5. Univariate Cox Regression analyzing the prognostic factors for osteosarcoma patients with distant metastases (between January 2010 and December 2014).

| **Subject characteristics** | **M-Met (231)** | | **Bone-Met (50)** | | **Brain-Met (6)** | | **Liver-Met (7)** | | **Lung-Met (189)** | |
| --- | --- | --- | --- | --- | --- | --- | --- | --- | --- | --- |
|  | **HR (95%CI)** | ***P-value*** | **HR (95%CI)** | ***P-value*** | **HR (95%CI)** | ***P-value*** | **HR (95%CI)** | ***P-value*** | **HR (95%CI)** | ***P-value*** |
| **Sex** |  |  |  |  |  |  |  |  |  |  |
| Male | 1.00 (Reference) |  | 1.00 (Reference) |  | 1.00 (Reference) |  | 1.00 (Reference) |  | 1.00 (Reference) |  |
| Female | 0.87 (0.62-1.21) | 0.399 | 1.26 (0.68-2.37) | 0.462 | 0.03 (0.00-114.27) | 0.391 | 0.30 (0.03-2.65) | 0.276 | 0.85 (0.58-1.24) | 0.392 |
| **Age** |  |  |  |  |  |  |  |  |  |  |
| 0-24 | 1.00 (Reference) |  | 1.00 (Reference) |  | 1.00 (Reference) |  | NA | NA | 1.00 (Reference) |  |
| 25-59 | 2.05 (1.38-3.03) | <0.001 | 2.62 (1.18-5.81) | 0.018 | NA | NA | NA | NA | 2.16 (1.39-3.36) | 0.001 |
| ≥60 | 6.30 (4.26-9.32) | <0.001 | 7.44 (3.19-17.37) | <0.001 | 1.41 (0.08-23.57) | 0.809 | NA | NA | 6.12 (3.92-9.56) | <0.001 |
| **Race** |  |  |  |  |  |  |  |  |  |  |
| White | 1.00 (Reference) |  | 1.00 (Reference) |  | 1.00 (Reference) |  | 1.00 (Reference) |  | 1.00 (Reference) |  |
| Black | 0.76 (0.48-1.19) | 0.233 | 0.53 (0.21-1.33) | 0.176 | 4.61 (0.41-51.31) | 0.214 | 0.99 (0.10-9.42) | 0.994 | 0.78 (0.46-1.32) | 0.357 |
| Others | 0.88 (0.50-1.56) | 0.663 | NA | NA | NA | NA | 0.61 (0.06-5.92) | 0.673 | 0.84 (0.44-1.62) | 0.610 |
| Unknown | NA | NA | NA | NA | NA | NA | NA | NA | NA | NA |
| **Insurance recode** |  |  |  |  |  |  |  |  |  |  |
| Uninsured | 1.00 (Reference) |  | NA | NA | NA | NA | NA | NA | 1.00 (Reference) |  |
| Insured | 0.89 (0.33-2.40) | 0.817 | NA | NA | NA | NA | NA | NA | 0.80 (0.30-2.18) | 0.668 |
| Unknown | NA | NA | NA | NA | NA | NA | NA | NA | NA | NA |
| **Marital status** |  |  |  |  |  |  |  |  |  |  |
| Unmarried | 1.00 (Reference) |  | 1.00 (Reference) |  | NA | NA | 1.00 (Reference) | 1.00 | 1.00 (Reference) |  |
| Married | 2.56 (1.79-3.65) | <0.001 | 3.74 (1.86-7.53) | <0.001 | NA | NA | 0.36 (0.06-2.21) | 0.268 | 2.78 (1.85-4.18) | <0.001 |
| Unknown | NA | NA | NA | NA | NA | NA | NA | NA | NA | NA |
| **Primary site** |  |  |  |  |  |  |  |  |  |  |
| Extremity | 1.00 (Reference) |  | 1.00 (Reference) |  | 1.00 (Reference) |  | NA | NA | 1.00 (Reference) |  |
| Axial | 2.85 (2.01-4.04) | <0.001 | 2.93 (1.51-5.67) | 0.001 | 0.93 (0.15-5.80) | 0.938 | NA | NA | 2.93 (1.95-4.39) | <0.001 |
| Unknown | NA | NA | NA | NA | NA | NA | NA | NA | NA | NA |
| **Histology** |  |  |  |  |  |  |  |  |  |  |
| Osteosarcoma NOS | 1.00 (Reference) |  | 1.00 (Reference) |  | NA | NA | NA | NA | 1.00 (Reference) |  |
| Chondroblastic | 0.59 (0.36-0.98) | 0.043 | 0.39 (0.09-1.62) | 0.193 | NA | NA | NA | NA | 0.63 (0.36-1.11) | 0.111 |
| Central | 0.37 (0.12-1.17) | 0.089 | NA | NA | NA | NA | NA | NA | 0.34 (0.08-1.36) | 0.126 |
| Parosteal | 0.82 (0.11-5.90) | 0.847 | NA | NA | NA | NA | NA | NA | 0.87 (0.12-6.26) | 0.890 |
| Fibroblastic | 0.61 (0.19-1.91) | 0.393 | 0.40 (0.05-2.94) | 0.367 | NA | NA | NA | NA | 0.66 (0.21-2.09) | 0.482 |
| Telangiectatic | NA | NA | NA | NA | NA | NA | NA | NA | NA | NA |
| Others | 1.63 (0.80-3.34) | 0.182 | 0.79 (0.28-2.25) | 0.661 | NA | NA | 0.62 (0.07-5.73) | 0.669 | 2.11 (0.97-4.59) | 0.059 |
| **Grade** |  |  |  |  |  |  |  |  |  |  |
| Grade I | NA | NA | NA | NA | NA | NA | NA | NA | NA | NA |
| Grade II | 1.00 (Reference) | 1.00 | NA | NA | NA | NA | NA | NA | 1.00 (Reference) | 1.00 |
| Grade III | 0.52 (0.19-1.45) | 0.209 | NA | NA | NA | NA | NA | NA | 0.51 (0.18-1.45) | 0.206 |
| Grade IV | 0.54 (0.20-1.49) | 0.233 | NA | NA | NA | NA | NA | NA | 0.51 (0.18-1.40) | 0.190 |
| Unknown | NA | NA | NA | NA | NA | NA | NA | NA | NA | NA |
| **T stage** |  |  |  |  |  |  |  |  |  |  |
| T1 | 1.00 (Reference) |  | 1.00 (Reference) |  | 1.00 (Reference) |  | NA | NA | 1.00 (Reference) |  |
| T2 | 1.10(0.71-1.71) | 0.666 | 0.28(0.11-0.67) | 0.005 | 0.50(0.03-8.03) | 0.625 | NA | NA | 1.10(0.65-1.86) | 0.713 |
| T3 | 1.40(0.68-2.90) | 0.365 | 0.89(0.29-2.71) | 0.832 | 0.00(0.00-94.97) | 0.290 | NA | NA | 1.38(0.62-3.08) | 0.429 |
| Unknown | NA | NA | NA | NA | NA | NA | NA | NA | NA | NA |
| **N stage** |  |  |  |  |  |  |  |  |  |  |
| N0 | 1.00 (Reference) |  | 1.00 (Reference) |  | 1.00 (Reference) |  | 1.00 (Reference) |  | 1.00 (Reference) |  |
| N1 | 1.73(0.93-3.21) | 0.084 | 2.35(0.68-8.14) | 0.180 | 4.61(0.41-51.31) | 0.214 | 1.56(0.14-17.75) | 0.719 | 1.69(0.82-3.49) | 0.156 |
| Unknown | NA | NA | NA | NA | NA | NA | NA | NA | NA | NA |
| **Number of mets** |  |  |  |  |  |  |  |  |  |  |
| ≤1 | 1.00 (Reference) |  | 1.00 (Reference) |  | 1.00 (Reference) |  | 1.00 (Reference) |  | 1.00 (Reference) |  |
| ＞1 | 2.33 (0.57-9.47) | 0.237 | 1.52 (0.36-6.35) | 0.569 | 0.03 (0.00-114.27) | 0.391 | 0.62 (0.07-5.73) | 0.669 | 2.33 (0.57-9.47) | 0.237 |
| **Surg (prim)** |  |  |  |  |  |  |  |  |  |  |
| None | 1.00 (Reference) |  | 1.00 (Reference) |  | 1.00 (Reference) |  | 1.00 (Reference) |  | 1.00 (Reference) |  |
| Yes | 0.27 (0.19-0.37) | <0.001 | 0.27 (0.14-0.54) | <0.001 | 1.12 (0.11-11.18) | 0.920 | 0.03 (0.00-102.21) | 0.390 | 0.27 (0.19-0.40) | <0.001 |
| Unknown | NA | NA | NA | NA | NA | NA | NA | NA | NA | NA |

Abbreviations: Met=Metastases.
