# Supplementary material for: Risk and Prognostic Factors for Different Organ Metastasis in Primary Osteosarcoma: A Large Population‐Based Analysis
Source: Orthop Surg. 2022 Mar 16;14(4):714–9. doi: 10.1111/os.13243 (PMC9002071; doi:10.1111/os.13243)
Supplement: Supplementary file 6 — Supplementary Table S6 Multivariate Cox regression analyzing the prognostic factors of osteosarcoma patients with distant metastases (diagnosed between 2010 and 2014) [file OS-14-714-s003.docx]

Supplementary table S6. Multivariate Cox Regression analyzing the prognostic factors of osteosarcoma patients with distant metastases (diagnosed between 2010 and 2014).

| **Subject characteristics** | **M-Met (231)** | | **Bone-Met (50)** | | **Lung-Met (189)** | |
| --- | --- | --- | --- | --- | --- | --- |
|  | **HR (95%CI)** | ***P-value*** | **HR (95%CI)** | ***P-value*** | **HR (95%CI)** | ***P-value*** |
| **Sex** |  |  |  |  |  |  |
| Male | 1.00 (Reference) |  | 1.00 (Reference) |  | 1.00 (Reference) |  |
| Female | 0.94 (0.60-1.48) | 0.795 | 3.85 (1.02-14.51) | 0.047 | 0.77 (0.46-1.31) | 0.339 |
| **Age** |  |  |  |  |  |  |
| 0-24 | 1.00 (Reference) |  | 1.00 (Reference) |  | 1.00 (Reference) |  |
| 25-59 | 1.96 (1.11-3.45) | 0.020 | 3.06 (0.54-17.33) | 0.207 | 1.60 (0.85-3.01) | 0.145 |
| ≥60 | 3.97 (1.99-7.91) | <0.001 | 0.62 (0.09-4.10) | 0.620 | 3.07 (1.37-6.88) | 0.007 |
| **Primary site** |  |  |  |  |  |  |
| Extremity | NA | NA | 1.00 (Reference) |  | NA | NA |
| Axial | NA | NA | 1.00 (0.26-3.91) | 0.998 | NA | NA |
| Unknown | NA | NA | NA | NA | NA | NA |
| **Histology** |  |  |  |  |  |  |
| Osteosarcoma NOS | 1.00 (Reference) |  | NA | NA | 1.00 (Reference) |  |
| Chondroblastic | 0.82 (0.43-1.58) | 0.559 | NA | NA | 0.80 (0.39-1.65) | 0.550 |
| Central | 0.54 (0.16-1.78) | 0.311 | NA | NA | 0.37 (0.12-1.17) | 0.413 |
| Parosteal | 0.79 (0.09-7.38) | 0.838 | NA | NA | 0.82 (0.11-5.90) | 0.867 |
| Fibroblastic | 0.71 (0.21-2.39) | 0.586 | NA | NA | 0.78 (0.23-2.65) | 0.696 |
| Telangiectatic | NA | NA | NA | NA | NA | NA |
| Others | 1.01 (0.43-2.33) | 0.987 | NA | NA | 1.26 (0.49-3.25) | 0.632 |
| **T stage** |  |  |  |  |  |  |
| T1 | 1.00 (Reference) |  | 1.00 (Reference) |  | 1.00 (Reference) |  |
| T2 | 0.95 (0.58-1.57) | 0.850 | 0.06 (0.01-0.47) | 0.007 | 0.92 (0.51-1.67) | 0.781 |
| T3 | 1.18 (0.54-2.58) | 0.684 | 0.15 (0.02-1.55) | 0.112 | 1.10 (0.45-2.74) | 0.830 |
| Unknown | NA | NA | NA | NA | NA | NA |
| **N stage** |  |  |  |  |  |  |
| N0 | 1.00 (Reference) |  | 1.00 (Reference) |  | 1.00 (Reference) |  |
| N1 | 0.75 (0.34-1.66) | 0.470 | 3.67(0.34-38.96) | 0.281 | 0.78 (0.31-1.95) | 0.601 |
| Unknown | NA | NA | NA | NA | NA | NA |
| **Number of mets** |  |  |  |  |  |  |
| ≤1 | 1.00 (Reference) |  | NA | NA | 1.00 (Reference) |  |
| ＞1 | 2.33 (0.57-9.47) | 0.237 | NA | NA | 2.33 (0.57-9.47) | 0.237 |
| **Surg (prim)** |  |  |  |  |  |  |
| None | 1.00 (Reference) |  | 1.00 (Reference) |  | 1.00 (Reference) |  |
| Yes | 0.41 (0.25-0.69) | 0.001 | 0.56 (0.17-1.80) | 0.331 | 0.30 (0.16-0.57) | <0.001 |
| Unknown | NA | NA | NA | NA | NA | NA |

Abbreviations: Met=Metastases.
